# Research on New and Traditional Energy Sources in OECD Countries

**DOI:** 10.3390/ijerph16071122

**Published:** 2019-03-28

**Authors:** Ying Li, Yung-ho Chiu, Tai-Yu Lin

**Affiliations:** 1 Business School, Sichuan University, Wangjiang Road No. 29, Chengdu 610064, China; liyinggs@scu.edu.cn; 2 Department of Economics, Soochow University, 56, Kueiyang St., Sec. 1, Taipei 10048, Taiwan; eickyla@gmail.com

**Keywords:** dynamic DEA, new energy, OECD, PM_2.5_, undesirable output

## Abstract

To mitigate the problems associated with climate change, the low-carbon economy concept is now being championed around the world in an effort to reduce greenhouse gas emissions and ensure sustainable economic growth. Therefore, to reduce the dependence on traditional energy sources, the Organization for Economic Co-operation and Development (OECD) has been actively promoting the use of renewable energy. Past research has tended to neglect the influence of other pollutants such as fine particulate matter (PM_2.5_) and sulfur dioxide (SO_2_) and have mainly been based on static analyses. To make up for these research gaps, this study examined OECD country data from 2010–2014, with labor, fixed assets, new energy, and traditional energy as the inputs, and Gross Domestic Product (GDP), carbon dioxide (CO_2_), and PM_2.5_ as the outputs, from which it was found: (1) the overall efficiency of the individual countries varied significantly, with nine countries being found to have efficiencies of 1 for all five years, but many others having efficiencies below 0.2; (2) in countries where there was a need for improvements in traditional energy (which here refers to coal, petroleum and other fossil energy sources), there was also a significant need for improvement in new energy sources (which here refers to clean energy which will produce pollutant emissions and can be directly used for production and life, including resources like nuclear energy and “renewable energy”); (3) countries with poor traditional energy and new energy efficiencies also had poor CO_2_ and PM_2.5_ efficiencies; (4) many OECD countries have made progress towards sustainable new energy developments

## 1. Introduction

As the demand for energy continues to grow, the levels of carbon dioxide emitted into the atmosphere have also been increasing, which is adversely affecting efforts to combat climate change. The UN Intergovernmental Panel on Climate Change (IPCC) has clearly stated that climate change is a reality and is continuing to intensify around the world. One of the main climate change causes has been man-made carbon emissions from the use of fossil fuel derived energy; therefore, if countries do not implement CO_2_ reduction targets, global carbon dioxide will continue to increase. As the pollution caused by traditional petrochemical energy has increased year on year, many countries are seeking energy generation alternatives, and have begun to invest in new energy developments with the aim of future sustainable development.

New energy research has focused on the impact of new energy on GDP or CO_2_ levels [1,2,3,4,5,6,7,8,9,10,11,12,13,14,15,16,17,18,19,20,21,22,23,24,25,26,27,28,29,30,31,32], with most evaluations having been conducted using DEA methods such as CCR, BCC, SBM, Distance functions and two-stage DEA analysis. However, previous analyses in this area have tended to employ only static analyses that did not reveal the continuous development of new energy. Further, most analyses have only considered CO_2_ and have ignored the comprehensive consideration of other air pollution indicators such as PM_2.5_. Therefore, to resolve these shortcomings, this paper uses a modifies Dynamic DEA model to explore OECD new energy efficiency and sustainability. This model uses descriptive statistics and dynamic DEA to analyze annual input and output and efficiency performance of OECD countries during 2010–2014. Comparing the differences in annual energy efficiency indicators of traditional energy, new energy, CO_2_ and PM_2.5_ in OECD countries, this paper provides insight into the performance of OECD countries in terms of overall efficiency, traditional energy efficiency, new energy efficiency, CO_2_ and PM_2.5_ emission efficiency, and this research makes policy suggestions. The contribution of this paper is to present a dynamic analysis that also considers PM_2.5_ to comprehensively assess the efficiency of new energy, traditional energy, and CO_2_ and PM_2.5_ in OECD countries from 2010–2014, with labor, fixed assets, new energy, and traditional energy consumption being the inputs, GDP being the desirable output, and CO_2_ and PM_2.5_ being the undesirable outputs.

The remainder of this paper is organized as follows: Section 2 gives a literature review, Section 3 comprehensively describes the research method, Section 4 gives the empirical results and discussion, and Section 5 presents the conclusions and policy proposals.

## 2. Literature Review

Past new energy research has taken three main directions; the impact of new energy on GDP or CO_2_, the sustainable development of the environment, or new energy efficiency and government policies. To analyze the impact of new energy on GDP and CO_2_, Bampatsou et al. [1] used a technical efficiency index from 15 EU countries from 1980 to 2008, and found that of the total energy input level, the nuclear energy input had a negative impact on technical efficiency, and when the consumption of fossil fuels was reduced and the renewable energy was better utilized, output significantly increased. Komiyama and Fujii [2] examined whether solar energy could be integrated into Japan’s power system to its optimize power generation structure, finding that solar energy could improve Japan’s economic performance. Pang et al. [3] examined economic output, energy conservation, emissions reduction, and the impact of clean energy use on total factor efficiency for 87 countries from 2004–2010, and found that clean energy consumption significantly increased the total-factor emissions reduction efficiency (TFCE), slightly improved total-factor economic output efficiency (TFYE), and significantly reduced total-factor energy efficiency (TFEE). Adewuyi and Awodumi [4] suggested that the pollution effects of the energy-growth nexus should be considered in sustainable development policies. Jebali et al. [5] used a double bootstrap DEA and bias correction efficiency truncated regression to study the energy efficiency of Mediterranean countries from 2009 to 2012. It is found that even though these countries had high energy efficiency levels, it had declined over time due to the effects of per capita GNI, population density, and renewable energy use. Atem and Hotaling [6] used System Generalized Method of Moments (GMM) data from 174 countries from 1980 and 2012 to analyze the impact of power generation on economic growth. They found that there was a significant relationship between renewable energy and non-renewable energy power generation and economic growth.

In sustainable development research, Hoang and Rao [7] used a non-radial DEA to analyze energy efficiency in 29 OECD countries, and found that agricultural production systems were generally sustainable, and Shiau and Jhang [8] used a Radial DEA to analyze the efficiency of Taiwan’s transportation system, finding that when the three core service impact indicators, cost efficiency, and service reductions were excellent, the transportation system was sustainable. Camioto et al. [9] used an SBM DEA to analyze the overall efficiency of various industries in Brazil and they found that the most efficient industry in Brazil was the textile industry, and that the sustainable development of the metallurgical industry was poor. Wang [10] used an SBM DEA to analyze the energy efficiency of 109 countries, finding that high-income countries had more sustainable energy than low- and middle-income countries. Zhang and Xie [11] used a non-radial DDF to explore China’s renewable energy and sustainable development from 1991 to 2005. It is found that China’s environmental supervision costs needed to be increased to improve renewable energy efficiency. Raheli et al. [12] surveyed the Marinde region of Azerbaijan Province in eastern Iran to assess the sustainability and efficiency of tomato production using a fractional regression model (FRM) for analysis of the first stage, and farm-specific variables such as education level, farmer age, total land area, and manure in the second stage, and found that farmer age and education level as well as area had an impact on tomato production efficiency.

In energy efficiency and government policy analysis research, Chien and Ho [13] used a radial DEA to analyze the overall economic efficiency of 45 OECD countries. It is found that an increase in renewable energy could increase economic efficiency. Homma and Hu [14] studied the energy efficiency of 47 metropolitan areas in Japan from 1993 to 2003. They found that because of the high cost of renewable energy, the government needed to encourage these inefficient regions to change their industrial structures and reduce energy consumption to maintain the environment. Sueyoshi and Goto [15] used a non-radial DEA model to study the effect of the US Clean Air Act (CAA) on acid-induced gases (NO_x_), and concluded that the CAA had been effective in controlling SO_2_ and NO_2_ emissions from US coal-fired power plants. Blokhuis et al. [16] used radial DEA to analyze the efficiency of new energy in The Netherlands, and it is found that wind energy was able to improve energy technology efficiency. Boubaker [17] used radial DEA to analyze the energy efficiency of Morocco, Algeria and Tunisia, and found that energy diversification was more efficient and better suited the common interests of the three countries. Fagiani et al. [18] found that renewable energy was able to effectively reduce CO_2_ emissions from the power sector as well as reduce a country’s dependence on imported oil. Menegak and Gurluk [19] compared renewable energy in Turkey and Greece, and found that Greece’s poor performance was mainly due to its economic crisis, which had delayed the development of its renewable energy sector. Sueyoshi and Goto [20] used static DEA and dynamic Malmquist indices to assess the relationship between fuel mixtures, electricity and carbon dioxide in 10 industrial countries, finding that nuclear power generation in France and water and renewable energy in The Netherlands were important for the sustainable development of each society. Azamade et al. [21] used fuzzy-DEA to study Iranian wind power plants, and they found that they significantly contributed to energy consumption. Sueyoshi and Goto [22] evaluated the performance of 160 photovoltaic power plants in Germany and the United States, finding that the German power plants had more efficient operations. Hampf and Rodseth [23] proposed a new efficiency approach to analyze the impact of new energy policies on 160 asphalt-fired generating units in which the average generator set electric-to-carbon ratio was lower than the optimum ratio of 15.3%, which was because of non-autonomous reductions rather than management inefficiencies. Kim et al. [24] evaluated the efficiency of investing in three NRE technologies; wind power, photovoltaic power and fuel cells; finding that wind power was the most efficient.

Sueyoshi and Goto [25] assessed Japan’s energy efficiency, finding that nuclear power generation efficiency ranged from 10.4% to 13.7%, and water and gas power generation efficiency ranged from 22.4% to 40.5%. Wu et al. [26] used two-stage DEA to explore the performance of China’s production efficiency (first stage) and treatment efficiency (second stage), and found that the western and eastern regions needed to develop clean energy sources to achieve sustainable environmental development. Lin and Li [27] studied the relationship between coal-fired power generation and the coal consumption rate, and found that an increase in power generation hours would reduce the coal consumption rate. Ervured et al. [28] analyzed the renewable energy efficiency of the Turkish provinces, concluding that to improve overall technical efficiency in the future, investment decisions needed to be made for the inefficient regions. Guo et al. [29] found that energy savings and pollutant reduction required new technologies to ensure continued economic development in China. Steimberg et al. [31] suggested that the use of natural gas generator sets to replace coal-fired generating system would effectively reduce carbon dioxide emissions. Llamas et al. [32] analyzed the efficiency of a Mexican thermoelectric symbiosis plant and estimated its possible energy contribution by 2030. The above literature in the discussion of new energy is about the impact of the introduction of new energy on economic growth and CO_2_. The results show that the introduction of new energy will increase economic growth and reduce CO_2_ environmental pollution. The analysis of new energy efficiency and new energy policies, the main literature uses static DEA (such as radial, non-radial, direction distance, two stage) to explore that whether the introduction of new energy efficiency and the adoption of new energy policy can improve economic, energy and operational efficiency. The results show that new energy input increases economic, energy and operational efficiency and reduces CO_2_, SO_2_ and NO_2_ pollutants. The government should actively promote new energy. The discussion of new energy mainly depends on the impact of new energy on economic growth and the reduction of CO_2_ pollution or the introduction of new energy, and assesses its economy, new energy efficiency and energy consumption efficiency. The methods are based on static analysis and fail to consider new energy with traditional energy consumption. The air pollutants are CO_2_ and SO_2_ without PM_2.5_.Unfortunately, most of the above research was not dynamic analysis and did not consider other air pollution variables such as PM_2.5_ and SO_2_. Therefore, to overcome these omissions, this paper considers a PM_2._ environmental variable and employs a modified Dynamic DEA model to explore OECD new energy efficiencies.

## 3. Methodology

### 3.1. Dynamic DEA

Farrell [33] first proposed a production frontier that was applicable only for a single input and a single output. Charnes et al. [34] then proposed a multiple input and output data envelope model (CCR model), after which Banker et al. [35] proposed the BCC model that replaced the constant return to scale (CRS) assumption with a variable return to scale (VRS). Tone [36] then developed non-radial and non-oriented estimation method, the slacks based measure model (SBM model), to resolve the inequality problems associated with the input and outputs

In terms of dynamic DEA development, Kloop [37] developed window analysis, and Malmquist [38] and Fare et al. [39] designed the Malmquist index. However, these models did not consider intertemporal continuation activities and were not suitable for long-term efficiency measurements.

Fare and Grosskopf [40] used a carry-over to enable link variables in the dynamic models, which was subsequently further modified by Bogetoft et al. [41], Chen [42], Nemoto and Goto [43], Park and Park. [44], Chang et al. [45] and Sueyoshhi and Sekitani [46] Tone and Tsutsui [47] then designed four types of carryover variables for a Slacks-Based Measures (SBM) D-DEA model; (1) desirable (good), (2) undesirable (bad) (3) discretionary (free) and (4) non-discretionary(fixed); and the DEA model into three forms; input-oriented, output-oriented, and non-oriented; and then used their SBM model to find the optimal solution.

n DMUs (*j* = 1, …, *n*) over *t* periods (*t* = 1, …, T), where each firm has m inputs (*i* = 1, …, *m*), where *p* is the fixed inputs (*i* = 1, …, *p*), *s* is the output (*i* = 1, …, *s*), and *r* is the fixed output (*i* = 1, …, *r*). Each DMU has inputs and outputs in *t* period, with the carry-over (link) to the next period being t + 1. 

$x_{ijt}\left(i=1,\ldots ,m\right)$, $x_{ijt}^{fix}\left(i=1,\ldots ,p\right)$, $y_{itj}\left(i=1,\ldots ,s\right)$, $y_{it}^{fix}\left(i=1,\ldots ,r\right)$ indicates the desirable, undesirable, flexible, and fixed values for $DMU_{j}$ in t, and $z^{good}$, $z^{bad}$, $z^{free}$, $z^{fix}$ are the period activities.

Non-oriented model:

(1) $$\rho_{0}^{*}=\frac{\frac{1}{T}\sum_{t=1}^{T}W^{T}[1-\frac{1}{m+nbad}(\sum_{i=1}^{m}\frac{w_{i}^{-}s_{it}^{-}}{x_{iot}}+\sum_{i=1}^{nbad}\frac{s_{it}^{bad}}{z_{iot}^{bad}})}{\frac{1}{T}\sum_{t=1}^{T}W^{T}[1+\frac{1}{s+ngood}(\sum_{i=1}^{s}\frac{w_{i}^{+}s_{it}^{+}}{y_{iot}}+\sum_{i=1}^{ngood}\frac{s_{it}^{good}}{z_{iot}^{good}})}$$

(2) $$\overset{n}{\underset{j=1}{\sum }}z_{ijt}^{\alpha }\lambda_{j}^{t}=\overset{n}{\underset{j=1}{\sum }}z_{ijt}^{\alpha }\lambda_{j}^{t+1}\left(\forall i;t=1,\ldots ,T-1\right)$$

Equation (2) is the link equation for *t* and *t* + 1

(3) $$\begin{array}{c} x_{\mathrm{iot}}=\sum_{j=1}^{n}x_{\mathrm{ijt}}\lambda_{j}^{t}+s_{\mathrm{it}}^{-}\left(i=1,\ldots ,m;t=1,\ldots ,T\right)\\ x_{\mathrm{iot}}^{\mathrm{fix}}=\sum_{j=1}^{n}x_{\mathrm{iot}}^{\mathrm{fix}}\lambda_{j}^{t}\left(i=1,\ldots ,p;t=1,\ldots ,T\right)\\ y_{\mathrm{iot}}=\sum_{j=1}^{n}y_{\mathrm{ijt}}\lambda_{j}^{t}-s_{\mathrm{it}}^{+}\;\left(i=1,\ldots ,s;t=1,\ldots ,T\right)\\ y_{\mathrm{iot}}^{\mathrm{fix}}=\sum_{j=1}^{n}y_{\mathrm{iot}}^{\mathrm{fix}}\lambda_{j}^{t}\;\left(i=1,\ldots ,r;t=1,\ldots ,T\right)\\ z_{\mathrm{iot}}^{\mathrm{good}}=\sum_{j=1}^{n}z_{\mathrm{iot}}^{\mathrm{good}}\lambda_{j}^{t}-s_{\mathrm{it}}^{\mathrm{good}}\left(i=1,\ldots ,\mathrm{ngood};t=1,\ldots ,T\right)\\ z_{\mathrm{iot}}^{\mathrm{bad}}=\sum_{j=1}^{n}z_{\mathrm{ijt}}^{\mathrm{bad}}\lambda_{j}^{t}+s_{\mathrm{it}}^{\mathrm{bad}}\left(i=1,\ldots ,\mathrm{nbad};t=1,\ldots ,T\right)\\ z_{\mathrm{iot}}^{\mathrm{free}}=\sum_{j=1}^{n}z_{\mathrm{ijt}}^{\mathrm{free}}\lambda_{j}^{t}+s_{\mathrm{it}}^{\mathrm{free}}\left(i=1,\ldots ,\mathrm{nfree};t=1,\ldots ,T\right)\\ z_{\mathrm{iot}}^{\mathrm{fix}}=\sum_{j=1}^{n}z_{\mathrm{ijt}}^{\mathrm{fix}}\lambda_{j}^{t}\left(i=1,\ldots ,\mathrm{nfix};t=1,\ldots ,T\right)\\ \sum_{j=1}^{n}\lambda_{j}^{t}=1\left(t=1,\ldots ,T\right) \end{array}$$

(4) $$\lambda_{j}^{t}\geq 0,s_{it}^{-}\geq 0,s_{it}^{+}\geq 0,s_{it}^{good}\geq 0,s_{it}^{bad}\geq 0\;and\;s_{it}^{free}:free\left(\forall i,t\right)$$

The optimal model as the follows:

(5) $$\rho_{0t}=\frac{1-\frac{1}{m+nbad}\left(\sum_{i=1}^{m}\frac{w_{i}^{-}s_{iot}^{-*}}{x_{iot}}+\sum_{i=1}^{nbad}\frac{s_{iot}^{bad*}}{z_{iot}^{bad}}\right)}{1+\frac{1}{s+ngood}\left(\sum_{i=1}^{s}\frac{w_{i}^{+}s_{it}^{+*}}{y_{iot}}+\sum_{i=1}^{ngood}\frac{s_{iot}^{good*}}{z_{iot}^{good}}\right)}\left(i=1,\ldots ,T\right).$$

### 3.2. Modified Dynamic DEA Model

About DEA analysis on energy and environmental pollution efficiency, the main method is to compare static analysis. As this paper considers undesirable output in the dynamic SBM model, Tone and Tsutsui’s [47] dynamic SBM model was modified to include undesirable output. Thus, the Modified dynamic DEA model is as follows: Suppose the observation is a *J* (*J* = 1….*n*) dimension decision making unit (DMU) set in which the DMU under evaluation is represented by *DMU_o_* and is subject to *DMU_o_* ∈ *J*. The inputs and outputs used to compute the efficiency are labeled m inputs $x_{ijt}$ (*i* = 1…*m*) and s outputs Y*_ljt_*, respectively. Let output Y be divided into (Y^g^, Y^b^), where Y^g^ is the desirable output, Y^b^ is the undesirable output, and *Z^good^* is carried over from period t to period *t* + 1. The following is the non-oriented model:

(6) $$\theta_{0}^{*}=\min\frac{\frac{1}{T}\sum_{t=1}^{T}W^{t}\left[1-\frac{1}{m}\sum_{i=1}^{m}\frac{s_{it}^{-}}{x_{iot}}\right]}{\frac{1}{T}\sum_{t=1}^{T}W^{t}\left[1+\frac{1}{s_{1}+s_{2}+ngood}\left[\sum_{l=1}^{s_{1}}\frac{s_{jt}^{+g}}{y_{lot}^{g}}+\sum_{l=1}^{s_{2}}\frac{s_{jt}^{-b}}{y_{lot}^{b}}+\sum_{r=1}^{ngood}\frac{s_{rt}^{good}}{z_{rot}^{good}}\right]\right]}$$

Equation (6) is the connection equation between *t* and *t* + 1.

$$\sum_{j=1}^{n}z_{\mathrm{ijt}}^{\alpha }\lambda_{j}^{t}=\sum_{j=1}^{n}z_{\mathrm{ijt}}^{\alpha }\lambda_{j}^{t+1}\;\;\;\;\;\;\left(\forall i;t=1,\ldots ,T-1\right)$$

$$x_{\mathrm{iot}}=\overset{m}{\underset{i=1}{\sum }}x_{ijt}\lambda_{i}^{t}+s_{it}^{-}\;\;\;\;\;\;\left(i=1,\ldots ,m;t=1,\ldots ,T\right)$$

(7) $$y_{\mathrm{lot}}=\overset{s1}{\underset{l=1}{\sum }}y_{lot}^{+g}\lambda_{j}^{t}-s_{\mathrm{lt}}^{+g}\;\;\;\;\;\;(l=1,\ldots ,s1;t=1,\ldots ,T)$$

$$y_{\mathrm{lot}}=\overset{s2}{\underset{l=1}{\sum }}y_{lot}^{-b}\lambda_{j}^{t}-s_{\mathrm{lt}}^{-b}\left(\;\;\;\;\;\;l=1,\ldots ,s2;t=1,\ldots ,T\right)$$

$$z_{\mathrm{rot}}^{\mathrm{good}}=\overset{\mathrm{ngood}}{\underset{r=1}{\sum }}z_{\mathrm{rot}}^{\mathrm{good}}\lambda_{j}^{t}-s_{\mathrm{rt}}^{\mathrm{good}}\;\left(R=1,\ldots ,\mathrm{ngood};t=1,\ldots ,T\right)$$

$$\overset{n}{\underset{j=1}{\sum }}\lambda_{j}^{t}=1\;\;\;\;\;\;\left(t=1,\ldots ,T\right)$$

$$\lambda_{j}^{t}\geq 0,s_{\mathrm{it}}^{-}\geq 0,s_{\mathrm{lt}}^{+g}\geq 0,,s_{\mathrm{lt}}^{-b}\geq 0,s_{\mathrm{rt}}^{\mathrm{good}}\geq 0,$$

The most efficient solution is:

(8) $$\rho_{0t}=\frac{1-\frac{1}{m}\left(\sum_{i=1}^{m}\frac{s_{\mathrm{iot}}^{-*}}{x_{\mathrm{iot}}}\right)}{1+\frac{1}{s1+s2+\mathrm{ngood}}\left(\sum_{l=1}^{s1}\frac{s_{\mathrm{jt}}^{+g*}}{y_{lot}}+\sum_{l=1}^{s2}\frac{s_{\mathrm{jt}}^{-b*}}{y_{lot}}+\sum_{r=1}^{\mathrm{ngood}}\frac{s_{\mathrm{rot}}^{\mathrm{good}*}}{z_{\mathrm{rot}}^{\mathrm{good}}}\right)}\;\;\;\;\;\;\;\;\;\left(i=1,\ldots ,T\right).$$

### 3.3. New Energy, Energy Consumption, $CO_{2}$, and PM_2.5_ Efficiency Indices

Hu and Wang’s [48] total-factor energy efficiency index was used to overcome any possible bias in the traditional energy efficiency indicators. For each evaluated country, the GDP, energy consumption, new energy, CO_2_ and PM_2.5_ efficiencies were calculated using Equations (9)–(13):

(9) $$GDP=\frac{\mathrm{Actual}\;\mathrm{GDP}\;\mathrm{desirable}\;\mathrm{output}\;\left(i,t\right)}{\mathrm{Target}\;\mathrm{GDP}\;\mathrm{desirable}\;\mathrm{output}\;\left(i,t\right)}$$

(10) $$E=\frac{\mathrm{Target}\;\mathrm{energy}\;\mathrm{input}\;\left(i,t\right)}{\mathrm{Actual}\;\mathrm{energy}\;\mathrm{input}\;\left(i,t\right)}$$

(11) $$NE=\frac{\mathrm{Target}\;\mathrm{new}\;\mathrm{energy}\;\mathrm{input}\;\left(i,t\right)}{\mathrm{Actual}\;\mathrm{new}\;\mathrm{energy}\;\mathrm{input}\;\left(i,t\right)}$$

(12) $$CO_{2}=\frac{\mathrm{Target}\;CO_{2}\;\mathrm{Undesirable}\;\mathrm{output}\;\left(i,t\right)}{\mathrm{Actual}\;CO_{2}\;\mathrm{Undesirable}\;\mathrm{output}\;\left(i,t\right)}$$

(13) $${\mathrm{PM}}_{2.5}=\frac{\mathrm{Target}\;\mathrm{PM}2.5\;\mathrm{Undesirable}\;\mathrm{output}\;\left(i,t\right)}{\mathrm{Actual}\;\mathrm{PM}2.5\;\mathrm{Undesirable}\;\mathrm{output}\;\left(i,t\right)}$$

If the target E and NE input equaled the actual input and the CO_2_ and PM_2.5_ undesirable outputs equaled the actual undesirable outputs, then the E, NE, CO_2_, and PM_2.5_ efficiencies equaled 1, which indicated overall efficiency. If the target E and NE input was less than the actual input and the CO_2_ and PM_2.5_ undesirable outputs were less than the actual undesirable outputs, then the E, NE, CO_2_, and PM_2.5_ efficiencies were less than 1, which indicated overall inefficiency.

If the target GDP desirable output was equal to the actual GDP desirable output, then the GDP efficiency equaled 1, which indicated overall efficiency. If the actual GDP desirable output was less than the target GDP desirable output, then the GDP efficiency was less than 1, which indicated overall inefficiency.

## 4. Empirical Analyses

### 4.1. Sources and Variables

#### 4.1.1. Data Sources

This study researched the energy use performance of OECD member countries from 2010 to 2014; the data for which were extracted from the world development indicators of the world bank [49] and the climate analysis indicators tool of the world resource institute (WRI). Currently, there are 35 member OECD countries, include the United Arab Emirates, Argentina, Brazil, Botswana, China, Colombia, Costa Rica, Dominican Republic, Algeria, Indonesia, India, Iran, Kenya, Cambodia, Sri Lanka, Morocco, Malaysia, Nigeria, Nepal, Pakistan, Peru, Philippines, Romania, Russia, Singapore, Thailand, and South Africa. The goal was to compare the energy use efficiencies of the OECD members and identify the differences between the old and new energy consumption efficiencies.

#### 4.1.2. Variables

Labor, old energy consumption, and new energy consumption were the inputs, GDP, CO_2_, and PM_2.5_ emissions were the outputs, and fixed assets was the carry-over. The variable details are shown in Table 1.

Inputs: labor (10,000 people): employed people over 15 years of age, the unemployed, and first-time job seekers.

Traditional energy consumption: fossil fuel consumption as a percentage of total energy consumption.

New energy consumption: renewable energy consumption as a percentage of total energy consumption.

Output: GDP (millions of US dollars): total market value of the final goods and services produced in a country in a certain period of time.

Carbon dioxide emissions (thousand tonnes): carbon dioxide emissions from the combustion of solid, liquid and gaseous fuels.

PM_2.5_ (micrograms per cubic meter): air particles with a particle size less than or equal to 2.5 microns.

Carry over: fixed asset (millions of US dollars): total capital formation (total domestic investment) including fixed asset expenditure and inventory changes.

### 4.2. Statistical Analysis

**Figure 1:**
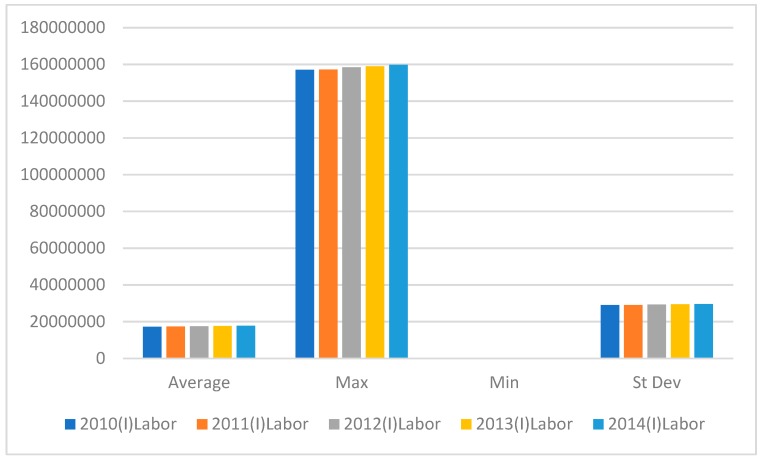
Labor Input variables from 2010–2014.

The following figures show the statistics for the input indicators (labor, capital, and new and old energy consumption) and the output indicators (gross domestic product). In Figure 1, the maximum labor force rose slowly. In 2012, there was a significant increase on the previous two years, after which the growth slowed. The average labor input in 2013 was slightly lower than in 2012, and the minimum labor force declined slightly in 2011 but then rose slowly from 2012 to 2014.

**Figure 2:**
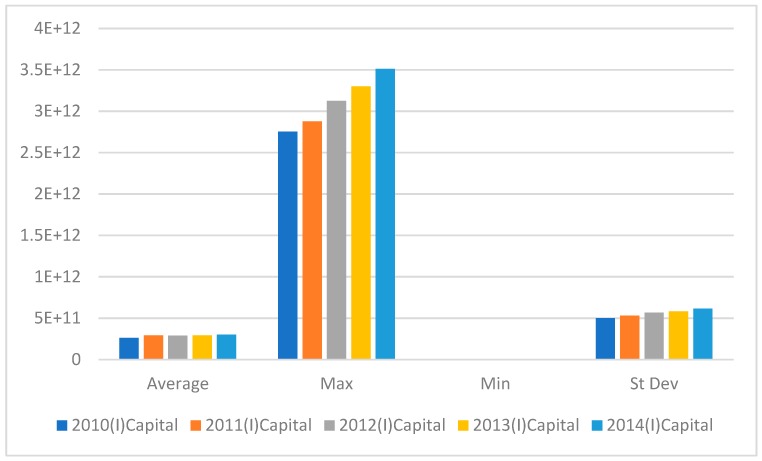
Fixed asset Input variables from 2010–2014.

Figure 2 shows that the maximum fixed assets rose substantially; however, the average value decreased in 2012 and then rose from 2013.

**Figure 3:**
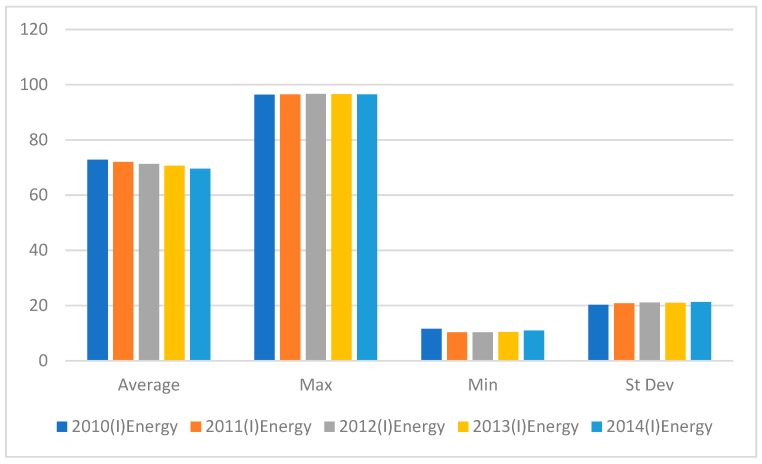
Energy Input variables from 2010–2014.

Figure 3 shows that the maximum value for traditional energy consumption fluctuated and the average value declined. In 2012, the maximum traditional energy consumption value was at its highest, after which there was a slow decline and a slight rebound in 2014; however, the level was lower than in 2012. Policy interventions were responsible for traditional energy consumption. The minimum traditional energy consumption value was in 2012, after which it increased slightly.

**Figure 4:**
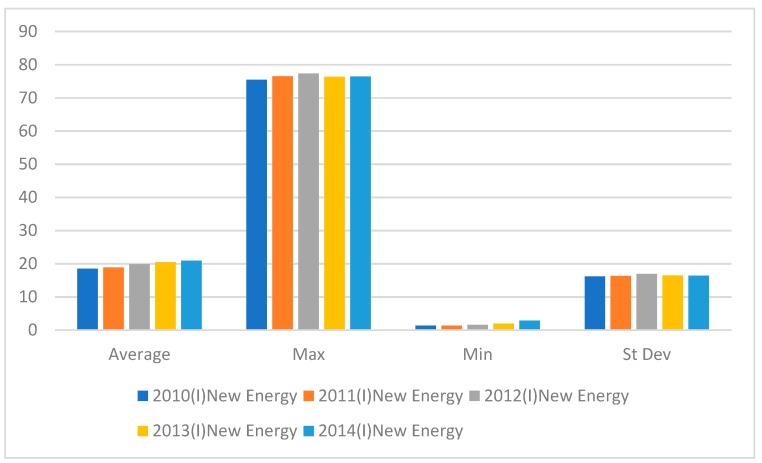
New Energy Input variables from 2010–2014.

Figure 4 shows that the maximum new energy consumption value was the highest in 2012, declined in 2013, and increased slightly in 2014. The minimum value rose sharply and reached its highest in 2014.

**Figure 5:**
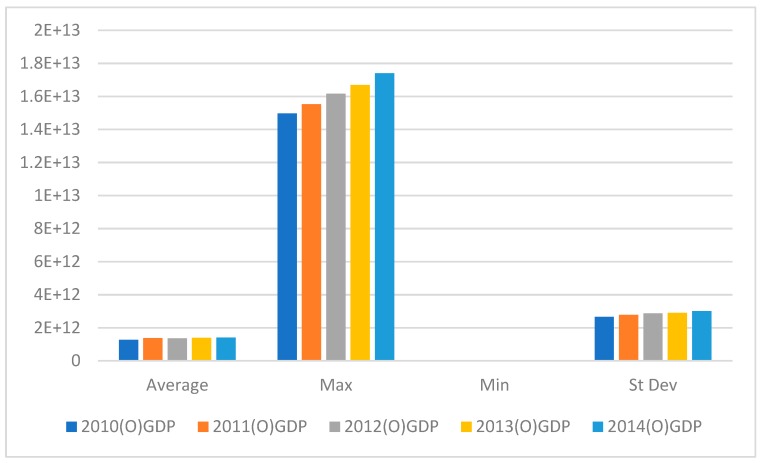
GDP output variables from 2010–2014.

Figure 5 shows that the maximum GDP value rose but the average value fluctuated, increasing in 2011, falling in 2012, increasing in 2013, and rising slightly in 2014.

### 4.3. Overall Efficiency and Efficiencies in Each Year

**Figure 6:**
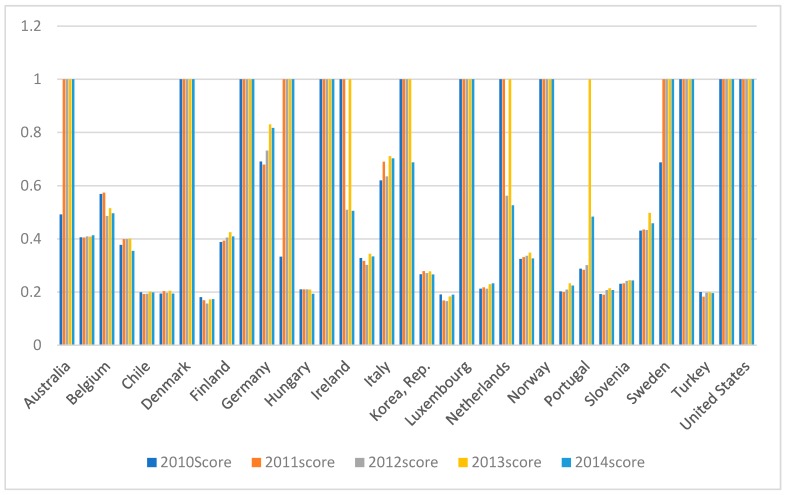
Overall and annual efficiencies by country.

Table 2 and Figure 6 show the overall efficiency and annual efficiency in each country. The countries that had efficiencies of in all five years were Australia, Denmark, France, Iceland, Luxembourg, Norway, Switzerland, United Kingdom, and the United States, countries with poor efficiencies at 0.2 or lower were Chile, Czech Republic, Estonia, Hungary, Latvia, Mexico, Poland, Slovak Republic, and Turkey with all other countries having efficiencies between 0.2 and 0.8.

Germany, Italy, Sweden, and Australia’s annual efficiencies all rose, with Sweden’s attaining 1. In 2014, Germany had an increase in annual efficiency, in 2013 and 2014, Italy’s efficiency fluctuated then rose to close to 0.7; however, Belgium, Canada, Ireland, the Netherlands, and Japan had some lower annual efficiencies, with the Netherlands having a significant decline in 2012 and 2014; however, its efficiency in all other years was 1. Japan’s efficiency declined in 2014, but the efficiency score was attained 1 in all other years.

### 4.4. Input and Output Efficiency

Table 3 and Table 4 show the efficiencies for labor and fixed assets from 2010 to 2014, from which it can be seen the gap between the countries for fixed-assets was smaller than for labor. Australia, Austria, Belgium, Demark, Finland, France, Iceland, Iceland, Ireland, Luxemburg, Netherlands, Switzerland all had labor efficiencies of 1, Canada, Germany, Israel, Italy, New Zealand and Sweden had efficiencies slightly less than 1, but above 0.9, and Chile, the Czech Republic, Estonia, Hungry, Korea Republic, Latvia, Mexico, Poland, Slovak Republic, Spain, Turkey had efficiencies below 0.7 for all years, with the countries needing the most improvement being Mexico, followed by Poland, Turkey and Latvia.

As shown in the Table 4, Canada, Chile, the Czech Republic, Estonia, Hungary, Korea Republic, Latvia, New Zealand, and Slovak republic all had fixed asset efficiencies below 0.8, Turkey had an efficiency of 1 in 2011 but below 0.7 in all other years, Spain’s efficiency in 2010 and 2011 was still below 0.7, but rose to above 0.8 in 2012, and nearly reached 0.9 in 2014, and Australia, Canada, Greece, Korea Republic, Mexico, Poland, Slovak Republic, Slovenia has rising fixed asset efficiencies.

Table 5 and Table 6 show that half of the countries had low scores for both new energy consumption and traditional energy consumption. Specifically, Australia, Demark, France, Iceland, Luxembourg, Norway, UK and the USA had traditional energy efficiencies of 1, Austria, Belgium, Canada, Chile, the Czech Republic, France, Hungary, Israel, Korea Republic, Latvia, Mexico, New Zealand, Poland, Slovak Republic, Slovenia, Turkey has traditional energy efficiencies below 0.2 for the five years, Finland’s traditional energy consumption efficiency was below 0.2 in 2010 and 2012, and rose to 0.2 in 2013, Germany’s traditional energy efficiency was around 0.8 for all five years, reached 0.9 in 2013, but declined in 2014, Italy’s traditional energy consumption efficiency rose from around 0.4 in 2010 to around 0.9 in 2014, the Netherlands’ was only 0.2 in 2012 and 2014, but was 1 in other years, Portugal reached 1 in 2013 but fell below 0.5 in 2014, Spain’s rose from around 0.3 in 2010 to around 0.45. in 2014, and Sweden’s rose from 0.6 in 2010 to 1 for the last four years.

There were three more countries; Australia, Greece, and Sweden; that had new energy consumption efficiencies of 1 than countries with traditional energy consumption efficiencies of 1, all of which had low traditional energy consumption efficiencies for one year. While Greece’s traditional energy consumption efficiency score in 2010 was less than 0.2, its new energy consumption efficiency was 1. Similarly, Sweden’s traditional energy consumption efficiency in 2010 was below 0.7, but its new energy consumption efficiency was 1. Japan’s new energy consumption efficiency was 0.95 in 2014 and 1 in other years, Italy’s was 0.6 in 2010, fell to nearly 0.2 in 2011, and began to rise slightly to around 0.4 in 2012, the Netherlands’ fell to 0.6 in 2012, was less than 0.5 in 2014, but was 1 in all other years, Portugal’s was 1 in 2013, and below 0.2 in 2010, 2011, and 2014, and Spain’s was only slightly higher than 0.2 in 2013, but in the other years, was lower than 0.2, and in 2011, was less than 0.1.

Austria, Canada, Chile, Czech Republic, Latvia, Estonia, Finland, Germany, Hungary, Israel, Italy, New Zealand, Slovak, Slovenia, and Turkey had both low traditional energy consumption efficiencies and low new energy consumption efficiencies. However, the new energy efficiency in some countries was better than their traditional energy efficiency; for example, Belgium had a new energy efficiency higher than 0.3 for five years, Korea’s efficiency was nearly 0.5 in 2010, but began to decline to be only slightly higher than 0.2 in 2014, which was still higher than its traditional energy efficiency, and the Netherlands had new energy efficiencies of 1 in 2011 and 2013, 0.6 in 2012, and slightly higher than 0.4 in 2014.

Table 7 and Table 8 show that the CO_2_ efficiencies in these five years were significantly higher than the PM_2.5_ efficiencies in almost all countries.

Belgium, Canada, Denmark, France, Germany, Iceland, Italy, Japan, Luxemburg, Netherlands, New Zealand, Spain, Sweden, Switzerland, United Kingdom, United States all had CO_2_ efficiencies of 1 in all five years, with the other 19 countries’ CO_2_ efficiencies being lower. The country with the lowest efficiency was Estonia, at only 0.2 for the five years; however, most other countries had efficiencies above 0.5.

Denmark, France, Iceland, Luxembourg, Norway, Switzerland, United Kingdom, and United States had PM_2.5_ efficiencies of 1 in all 5 years, Australia’s efficiency was only slightly higher than 0.4 in 2010 but was 1 in the other 4 years, Germany’s PM_2.5_ efficiency was slightly above 0.6 in 2010, fell to 0.6 in 2011, rose to above 0.7 in 2012 and then rose to above 0.8, Italy’s PM_2.5_ efficiency was between 0.5 and 0.6, and was slightly below 0.6 in 2014, Japan’s efficiency in the last year fell from 1 in the previous years to around 0.6, The Netherlands’ PM_2.5_ efficiency was 1 in 2010, 2011, and 2013, and was around 0.2 in 2012 and 2014, Portugal’s efficiency in 2011 and 2012 was around 0.1, was 1 in 2013 and fell to around 0.5 in 2014, and Spain’s efficiency increased from 0.4 in 2010 to 0.5 in 2013 and then fell slightly to just above 0.4 in 2014. Therefore, most countries needed to improve their PM_2.5_ efficiency. For example, Austria, Belgium, Canada, Chile, Czech Republic, Hungary, Israel, Korea Republic, Latvia, Mexico, New Zealand, Poland, Slovenia, Turkey all had PM_2.5_ efficiencies below 0.2, with many below 0.1. The efficiencies in Austria, Canada, Chile, France, Germany, Italy, New Zealand, Portugal, and Spain were generally rising, while the efficiencies in the Czech Republic, Estonia, Hungary, Korea, Latvia, Poland, Slovenia, and Turkey were decreasing.

To further understand the relationships between traditional energy and new energy and CO_2_ and PM_2.5_ in OECD countries, Table 9 was developed, from which it can be seen that the United States, United Kingdom, Switzerland, Denmark, France, Iceland, Luxembourg and Norway had traditional energy, new energy, CO_2_ and PM_2.5_, efficiencies of 1 for all 5 years, and that Australia, Germany, Greece, Japan, Ireland and Sweden Netherlands had poor performance for 1~2 years, but were efficient in most years. In Austria, Chile, the Czech Republic, Estonia, Finland, Hungary, Latvia, the Slovak Republic, Slovenia and Turkey, however, all indicators required improvement. Although the CO_2_ efficiencies in Belgium, Canada, Israel, Korea, Mexico, New Zealand and Poland were good, there was a need for significant improvements in traditional energy, new energy, and PM_2.5_.

In countries with both poor traditional energy and new energy efficiencies, the CO_2_ and PM_2.5_ efficiencies were also poor whereas the countries that had good new energy efficiencies, and especially the United States, Japan, and Western European countries, had moved toward the sustainable development of new energy.

## 5. Conclusions and Policy Suggestions

This paper analyzed the efficiency of new and traditional energy sources and CO_2_ and PM_2.45_ emissions, from which the following conclusions were made.

(1) There was a significant difference in the overall efficiencies in the OECD countries. Only Australia, Denmark, France, Iceland, Luxembourg, Norway, Switzerland, United Kingdom, and United States had overall efficiencies of 1 for the five years.
(2) However, Chile, the Czech Republic, Estonia, Hungary, Latvia, Mexico, Poland, Slovak Republic, and Turkey all had efficiencies around or below 0.2 for the five years, and therefore, had a significant need for improvement. As the efficiencies in the other 16 countries ranged from 0.2 to 0.8, there was some room for improvement.
(3) Germany, Italy, Sweden, and Australia had rising efficiencies and Belgium, Canada, Ireland, the Netherlands, and Japan had falling efficiencies. The Netherlands had a significant decline in 2012 and 2014, and efficiencies of 1 in all other years. Japan’s efficiency dropped in 2014, but was 1 in all other years.
(4) The differences in traditional energy efficiencies between the countries was large. Australia, Demark, France, Iceland, Luxembourg, Norway, UK and USA all had traditional energy efficiencies 1; however, Austria, Belgium, Canada, Chile, Czech Republic, France, Hungary, Israel, Korea Republic, Latvia, Mexico, New Zealand, Poland, Slovak Republic, Slovenia, and Turkey only had efficiencies below 0.2 for the five consecutive years. Obviously, there is a significant need for traditional energy efficiency improvements in many countries. Eleven countries have new energy efficiencies of 1, which was more than the countries with traditional energy efficiencies of 1. However, Austria, Canada, Chile, Czech Republic, Latvia, Estonia, Finland, Germany, Hungary, Israel, Italy, New Zealand, Slovak, Slovenia, and Turkey all had new energy efficiencies of less than 0.2 and had a significant need for improvements. Most Eastern European countries (Cezch, Hungary, Latvia, Poland, Lovenia etc..) had new energy efficiency scores below 0.10, and there was still much room for improvement.
(5) Belgium, Canada, Denmark, France, Germany, Iceland, Italy, Japan, Luxemburg, Netherlands, New Zealand, Spain, Sweden, Switzerland, United Kingdom, United States all had CO_2_ efficiencies of 1, and as the efficiencies in the other 19 countries were lower than 1, there was some need for improvement. Countries with relatively low carbon emission efficiency score were mainly Eastern European countries, Estonia only had an efficiency of 0.2 for the five years, while most other countries had efficiencies above 0.5.
(6) The PM_2.5_ efficiencies were generally much lower than the CO_2_ efficiencies, with only eight countries; Denmark, France, Iceland, Luxembourg, Norway, Switzerland, United Kingdom, and the United States; having efficiencies of 1 for the five years compared to 16 countries with CO_2_ efficiencies of 1. Austria, Belgium, Canada, Chile, Czech Republic, Hungary, Israel, Korea Republic, Latvia, Mexico, New Zealand, Poland, Slovenia, and Turkey all had PM_2.5_ efficiencies below 0.2, with most being lower that 0.1; therefore, there was a significant need for improvement. It could also be seen that the efficiency score for most Eastern European countries was also below 0.1. There was a lot of room for improvement.
(7) Countries that had poor traditional energy and new energy performances also had poor CO_2_ and PM_2.5_ performances.
(8) Although the new energy performance in many countries still needs to be strengthened, new energy has been performing better with many of the OECD countries moving towards the sustainable development of new energy.

Based on the above conclusions, most developed economies such as Australia, Denmark, France, Iceland, Luxembourg, Norway, Switzerland, United Kingdom, and the United States all had efficiencies of 1 for each indicator, and therefore there was no need for further improvements. While the Netherlands had a decline in new and traditional energy efficiencies in 2014, there is a greater need for efficiency improvements in Poland, the Czech Republic. Latvia, Hungary, the Slovak Republic, Slovenia, Israel, Turkey, New Zealand, Finland, and Spain, and some countries also need better energy consumption management and more effective environmental governance measures.

Eastern Europe was the most in need of an energy revolution to improve carbon efficiency and PM2.5 efficiency. For example, Poland’s economic development relies heavily on traditional energy, the coal industry. More than 80% of electricity in Poland comes from coal. In the future, it will increase the dependence on the production and consumption of coal to meet the needs of industrial development and residents’ lives. The health of Polish residents is greatly affected by the air pollution caused by coal burning. 44,000 people in the country die from air pollution every year and faces an increase in investment in disease management In the 50 cities with the most serious air pollution in the EU, 33 cities are in Poland. Poland has faced greater challenges in coping with carbon dioxide emissions and air pollutions around the world.

Measures that Eastern European countries, such as Poland, can take as follows:

1. Strengthening the development of new energy. The main new energy sources in Poland include offshore wind energy, nuclear energy and hydrogen energy. The development of new energy has become a top priority.
2. Industrial restructuring. Most of the economic growth in Eastern European countries depends on coal-based oil-based manufacturing, which brings large environmental pollution. The adjustment of industrial structure requires long-term government planning to reduce emissions of environmental pollutants.
3. Western Europe and Northern Europe have advanced technology and experience in environmental protection. International organizations should encourage developed countries provide technical assistance to Eastern European countries.
4. The Belt and Road Initiative provides conditions for cooperation between China and Eastern European countries. Use China’s air pollution control experience to strengthen China’s cooperation with these Eastern European countries.

OECD countries use resources efficiently by precisely controlling inputs and outputs in new energy. Unlike Western European countries and Nordic countries, Eastern European countries should pay attention to the treatment of air pollutant emissions and use resources on air pollutants reduction.

## Figures and Tables

**Table 1:** Input and output variables.

Input Variables	Desirable Output Variables	Undesirable Output Variables	Carry-Over
Labor (Lab)	GDP	CO_2_	Fixed assets (asset)
Energy consumption		PM_2.5_	
New energy consumption			

**Table 2:** Overall and annual efficiencies by country.

NO.	DMU	Total	2010	2011	2012	2013	2014
1	Australia	0.8830	0.4921	1.0000	1.0000	1.0000	1.0000
2	Austria	0.4079	0.4058	0.4043	0.4088	0.4077	0.4127
3	Belgium	0.5277	0.5690	0.5741	0.4860	0.5149	0.4957
4	Canada	0.3858	0.3773	0.3991	0.3989	0.4004	0.3543
5	Chile	0.1966	0.1987	0.1923	0.1931	0.2011	0.1977
6	Czech Republic	0.1986	0.1941	0.2032	0.1967	0.2051	0.1939
7	Denmark	1.0000	1.0000	1.0000	1.0000	1.0000	1.0000
8	Estonia	0.1703	0.1802	0.1690	0.1565	0.1724	0.1732
9	Finland	0.4038	0.3877	0.3928	0.4044	0.4245	0.4093
10	France	1.0000	1.0000	1.0000	1.0000	1.0000	1.0000
11	Germany	0.7480	0.6907	0.6792	0.7316	0.8299	0.8167
12	Greece	0.8285	0.3329	1.0000	1.0000	1.0000	1.0000
13	Hungary	0.2062	0.2098	0.2097	0.2095	0.2087	0.1932
14	Iceland	1.0000	1.0000	1.0000	1.0000	1.0000	1.0000
15	Ireland	0.7665	1.0000	1.0000	0.5094	1.0000	0.5054
16	Israel	0.3245	0.3274	0.3165	0.3019	0.3433	0.3339
17	Italy	0.6712	0.6193	0.6898	0.6346	0.7107	0.7027
18	Japan	0.9313	1.0000	1.0000	1.0000	1.0000	0.6879
19	Korea, Rep.	0.2718	0.2666	0.2780	0.2711	0.2779	0.2655
20	Latvia	0.1795	0.1907	0.1678	0.1658	0.1829	0.1901
21	Luxembourg	1.0000	1.0000	1.0000	1.0000	1.0000	1.0000
22	Mexico	0.2208	0.2119	0.2172	0.2126	0.2293	0.2327
23	Netherlands	0.7914	1.0000	1.0000	0.5618	1.0000	0.5258
24	New Zealand	0.3330	0.3243	0.3314	0.3358	0.3481	0.3257
25	Norway	1.0000	1.0000	1.0000	1.0000	1.0000	1.0000
26	Poland	0.2136	0.2019	0.2002	0.2091	0.2328	0.2237
27	Portugal	0.4404	0.2877	0.2834	0.3008	1.0000	0.4834
28	Slovak Republic	0.2020	0.1925	0.1896	0.2069	0.2142	0.2070
29	Slovenia	0.2382	0.2306	0.2322	0.2413	0.2440	0.2428
30	Spain	0.4502	0.4304	0.4350	0.4328	0.4973	0.4578
31	Sweden	0.9364	0.6874	1.0000	1.0000	1.0000	1.0000
32	Switzerland	1.0000	1.0000	1.0000	1.0000	1.0000	1.0000
33	Turkey	0.1946	0.1997	0.1825	0.1973	0.1985	0.1953
34	United Kingdom	1.0000	1.0000	1.0000	1.0000	1.0000	1.0000
35	United States	1.0000	1.0000	1.0000	1.0000	1.0000	1.0000

**Table 3:** Labor efficiency from 2010–2014.

DMU	2010 Labor	2011 Labor	2012 Labor	2013 Labor	2014 Labor
Australia	1.0000	1.0000	1.0000	1.0000	1.0000
Austria	1.0000	1.0000	1.0000	1.0000	1.0000
Belgium	1.0000	1.0000	1.0000	1.0000	1.0000
Canada	0.9144	0.9655	0.9493	0.9173	0.8416
Chile	0.3580	0.3745	0.3897	0.3926	0.3312
Czech Republic	0.4154	0.4411	0.4811	0.4718	0.4281
Denmark	1.0000	1.0000	1.0000	1.0000	1.0000
Estonia	0.3737	0.4140	0.4114	0.4426	0.4253
Finland	1.0000	1.0000	1.0000	1.0000	1.0000
France	1.0000	1.0000	1.0000	1.0000	1.0000
Germany	0.9678	0.9836	0.9429	0.9527	0.9426
Greece	0.7625	1.0000	1.0000	1.0000	1.0000
Hungary	0.3993	0.4008	0.3578	0.3684	0.3395
Iceland	1.0000	1.0000	1.0000	1.0000	1.0000
Ireland	1.0000	1.0000	1.0000	1.0000	1.0000
Israel	0.8765	0.9074	0.8638	0.9444	0.8886
Italy	1.0000	1.0000	0.9768	1.0000	0.9407
Japan	1.0000	1.0000	1.0000	1.0000	0.7143
Korea, Rep.	0.4546	0.4745	0.4603	0.4714	0.4782
Latvia	0.2931	0.3346	0.3300	0.3559	0.3420
Luxembourg	1.0000	1.0000	1.0000	1.0000	1.0000
Mexico	0.2182	0.2294	0.2180	0.2214	0.2175
Netherlands	1.0000	1.0000	1.0000	1.0000	1.0000
New Zealand	0.8269	0.8796	0.9117	0.9542	0.8928
Norway	1.0000	1.0000	1.0000	1.0000	1.0000
Poland	0.2788	0.2957	0.2691	0.2896	0.2723
Portugal	0.5004	0.5541	0.4955	1.0000	0.6299
Slovak Republic	0.4374	0.4492	0.4213	0.4345	0.4065
Slovenia	0.6037	0.6167	0.5574	0.5701	0.5384
Spain	0.7045	0.6780	0.6291	0.6546	0.6322
Sweden	0.9692	1.0000	1.0000	1.0000	1.0000
Switzerland	1.0000	1.0000	1.0000	1.0000	1.0000
Turkey	0.3212	0.3209	0.3203	0.3257	0.2997
United Kingdom	1.0000	1.0000	1.0000	1.0000	1.0000
United States	1.0000	1.0000	1.0000	1.0000	1.0000

**Table 4:** Fixed asset efficiency from 2010–2014.

DMU	2010 Asset	2011 Asset	2012 Asset	2013 Asset	2014 Asset
Australia	0.6857	1.0000	1.0000	1.0000	1.0000
Austria	0.9801	0.9812	0.9573	0.9413	0.9721
Belgium	0.9090	0.9287	1.0000	1.0000	0.9333
Canada	0.8047	0.8065	0.8134	0.8067	0.8205
Chile	0.6776	0.6314	0.5958	0.6383	0.7365
Czech Republic	0.6782	0.6879	0.6018	0.6632	0.6611
Denmark	1.0000	1.0000	1.0000	1.0000	1.0000
Estonia	0.7370	0.6197	0.5417	0.6079	0.6313
Finland	0.9296	1.0000	0.9555	1.0000	0.8922
France	1.0000	1.0000	1.0000	1.0000	1.0000
Germany	1.0000	1.0000	1.0000	1.0000	1.0000
Greece	0.9197	1.0000	1.0000	1.0000	1.0000
Hungary	0.7589	0.7606	0.8100	0.7772	0.7338
Iceland	1.0000	1.0000	1.0000	1.0000	1.0000
Ireland	1.0000	1.0000	1.0000	1.0000	1.0000
Israel	0.8502	0.7627	0.7420	0.8196	0.8468
Italy	1.0000	1.0000	1.0000	1.0000	1.0000
Japan	1.0000	1.0000	1.0000	1.0000	0.8044
Korea, Rep.	0.5744	0.5627	0.6242	0.6791	0.6894
Latvia	0.8116	0.6181	0.6020	0.6694	0.7478
Luxembourg	1.0000	1.0000	1.0000	1.0000	1.0000
Mexico	0.8340	0.8331	0.8395	0.9122	0.9344
Netherlands	1.0000	1.0000	1.0000	1.0000	1.0000
New Zealand	0.7808	0.7611	0.7531	0.7421	0.7576
Norway	1.0000	1.0000	1.0000	1.0000	1.0000
Poland	0.8631	0.8264	0.9219	1.0000	0.9911
Portugal	1.0000	0.8366	1.0000	1.0000	1.0000
Slovak Republic	0.6529	0.6232	0.7527	0.7796	0.7783
Slovenia	0.7050	0.7164	0.8424	0.8395	0.8737
Spain	0.9103	1.0000	1.0000	1.0000	0.9097
Sweden	0.9907	1.0000	1.0000	1.0000	1.0000
Switzerland	1.0000	1.0000	1.0000	1.0000	1.0000
Turkey	0.6820	1.0000	0.6837	0.6638	0.6952
United Kingdom	1.0000	1.0000	1.0000	1.0000	1.0000
United States	1.0000	1.0000	1.0000	1.0000	1.0000

**Table 5:** Traditional energy consumption efficiency score from 2010–2014.

No.	DMU	2010 Energy	2011 Energy	2012 Energy	2013 Energy	2014 Energy
1	Australia	0.1595	1.0000	1.0000	1.0000	1.0000
2	Austria	0.1442	0.1443	0.1590	0.1642	0.1677
3	Belgium	0.1812	0.2036	0.1827	0.1853	0.1489
4	Canada	0.1549	0.1898	0.1960	0.1714	0.1200
5	Chile	0.1016	0.1074	0.1219	0.1242	0.0972
6	Czech Republic	0.0147	0.0156	0.0864	0.0853	0.0761
7	Denmark	1.0000	1.0000	1.0000	1.0000	1.0000
8	Estonia	0.0342	0.0401	0.0376	0.0448	0.0495
9	Finland	0.0663	0.1423	0.0955	0.2169	0.2460
10	France	1.0000	1.0000	1.0000	1.0000	1.0000
11	Germany	0.7938	0.7861	0.8961	0.7934	0.8350
12	Greece	0.1195	1.0000	1.0000	1.0000	1.0000
13	Hungary	0.0636	0.0627	0.0571	0.0601	0.0563
14	Iceland	1.0000	1.0000	1.0000	1.0000	1.0000
15	Ireland	1.0000	1.0000	0.1349	1.0000	0.1428
16	Israel	0.0875	0.0885	0.0851	0.0927	0.0874
17	Italy	0.4500	0.5232	0.6819	0.8137	0.8859
18	Japan	1.0000	1.0000	1.0000	1.0000	0.6103
19	Korea, Rep.	0.0743	0.0784	0.0759	0.0771	0.0817
20	Latvia	0.0130	0.0146	0.0155	0.0158	0.0151
21	Luxembourg	1.0000	1.0000	1.0000	1.0000	1.0000
22	Mexico	0.0653	0.0698	0.0673	0.0692	0.0686
23	Netherlands	1.0000	1.0000	0.2224	1.0000	0.2040
24	New Zealand	0.0865	0.0920	0.0904	0.0958	0.0916
25	Norway	1.0000	1.0000	1.0000	1.0000	1.0000
26	Poland	0.0292	0.0310	0.0284	0.0624	0.0289
27	Portugal	0.0670	0.1056	0.1045	1.0000	0.4553
28	Slovak Republic	0.0460	0.0468	0.0447	0.0454	0.0432
29	Slovenia	0.0260	0.0258	0.0228	0.0231	0.0229
30	Spain	0.2872	0.3083	0.3654	0.4239	0.4436
31	Sweden	0.6647	1.0000	1.0000	1.0000	1.0000
32	Switzerland	1.0000	1.0000	1.0000	1.0000	1.0000
33	Turkey	0.0487	0.0499	0.0505	0.0535	0.0497
34	United Kingdom	1.0000	1.0000	1.0000	1.0000	1.0000
35	United States	1.0000	1.0000	1.0000	1.0000	1.0000

**Table 6:** New energy consumption efficiency from 2010–2014.

No.	DMU	2011 New Energy	2012 New Energy	2013 New Energy	2014 New Energy
1	Australia	1.0000	1.0000	1.0000	1.0000
2	Austria	0.0942	0.1054	0.1080	0.1132
3	Belgium	0.7831	0.3314	0.4552	0.4511
4	Canada	0.0467	0.0460	0.0935	0.0448
5	Chile	0.0150	0.0160	0.0199	0.0238
6	Czech Republic	0.0114	0.0337	0.0370	0.0393
7	Denmark	1.0000	1.0000	1.0000	1.0000
8	Estonia	0.0016	0.0017	0.0022	0.0025
9	Finland	0.0590	0.0294	0.0814	0.0682
10	France	1.0000	1.0000	1.0000	1.0000
11	Germany	0.2928	0.3352	0.7563	0.6759
12	Greece	1.0000	1.0000	1.0000	1.0000
13	Hungary	0.0249	0.0228	0.0285	0.0326
14	Iceland	1.0000	1.0000	1.0000	1.0000
15	Ireland	1.0000	0.5928	1.0000	0.6183
16	Israel	0.0496	0.0538	0.0662	0.0797
17	Italy	0.6014	0.2480	0.3997	0.4270
18	Japan	1.0000	1.0000	1.0000	0.9778
19	Korea, Rep.	0.4695	0.3977	0.3633	0.2548
20	Latvia	0.0014	0.0013	0.0016	0.0019
21	Luxembourg	1.0000	1.0000	1.0000	1.0000
22	Mexico	0.0680	0.0695	0.0731	0.0679
23	Netherlands	1.0000	0.6113	1.0000	0.4596
24	New Zealand	0.0090	0.0104	0.0135	0.0157
25	Norway	1.0000	1.0000	1.0000	1.0000
26	Poland	0.0266	0.0240	0.0417	0.0242
27	Portugal	0.0154	0.0218	1.0000	0.1895
28	Slovak Republic	0.0162	0.0162	0.0199	0.0201
29	Slovenia	0.0047	0.0042	0.0047	0.0053
30	Spain	0.1231	0.0932	0.2181	0.1628
31	Sweden	1.0000	1.0000	1.0000	1.0000
32	Switzerland	1.0000	1.0000	1.0000	1.0000
33	Turkey	0.0342	0.0357	0.0367	0.0413
34	United Kingdom	1.0000	1.0000	1.0000	1.0000
35	United States	1.0000	1.0000	1.0000	1.0000

**Table 7:** CO_2_ emissions efficiency from 2010–2014.

No.	DMU	2010 CO_2_	2011 CO_2_	2012 CO_2_	2013 CO_2_	2014 CO_2_
1	Australia	0.9758	1.0000	1.0000	1.0000	1.0000
2	Austria	0.7353	0.7215	0.7563	0.7616	0.7328
3	Belgium	1.0000	1.0000	1.0000	1.0000	1.0000
4	Canada	1.0000	1.0000	1.0000	1.0000	1.0000
5	Chile	0.6111	0.5442	0.5807	0.5595	0.4390
6	Czech Republic	0.6704	0.7268	0.3613	0.3549	0.2992
7	Denmark	1.0000	1.0000	1.0000	1.0000	1.0000
8	Estonia	0.2175	0.2129	0.2302	0.2113	0.1866
9	Finland	1.0000	0.5646	1.0000	0.6366	0.6681
10	France	1.0000	1.0000	1.0000	1.0000	1.0000
11	Germany	1.0000	1.0000	1.0000	1.0000	1.0000
12	Greece	0.7213	1.0000	1.0000	1.0000	1.0000
13	Hungary	0.5267	0.5030	0.5048	0.5367	0.4624
14	Iceland	1.0000	1.0000	1.0000	1.0000	1.0000
15	Ireland	1.0000	1.0000	0.7403	1.0000	0.5930
16	Israel	0.6852	0.6470	0.5996	0.7294	0.6630
17	Italy	1.0000	1.0000	1.0000	1.0000	0.9472
18	Japan	1.0000	1.0000	1.0000	1.0000	1.0000
19	Korea, Rep.	0.6963	0.6954	0.6636	0.6811	0.7261
20	Latvia	0.5944	0.6615	0.7008	0.7160	0.6256
21	Luxembourg	1.0000	1.0000	1.0000	1.0000	1.0000
22	Mexico	0.8163	0.8241	0.7576	0.7955	0.8167
23	Netherlands	1.0000	1.0000	1.0000	1.0000	1.0000
24	New Zealand	0.9318	0.9141	0.9081	0.9524	0.8041
25	Norway	1.0000	1.0000	1.0000	1.0000	1.0000
26	Poland	0.5464	0.5686	0.5286	0.4796	0.5763
27	Portugal	0.7056	0.8791	0.8375	1.0000	0.9650
28	Slovak Republic	0.4990	0.4861	0.5018	0.4978	0.4570
29	Slovenia	0.6326	0.5811	0.5520	0.5687	0.5410
30	Spain	1.0000	1.0000	1.0000	1.0000	1.0000
31	Sweden	1.0000	1.0000	1.0000	1.0000	1.0000
32	Switzerland	1.0000	1.0000	1.0000	1.0000	1.0000
33	Turkey	0.9339	0.8845	0.8404	0.9047	0.8157
34	United Kingdom	1.0000	1.0000	1.0000	1.0000	1.0000
35	United States	1.0000	1.0000	1.0000	1.0000	1.0000

**Table 8:** PM_2.5_ emissions efficiency from 2010–2014.

No.	DMU	2010 PM_2.5_	2011 PM_2.5_	2012 PM_2.5_	2013 PM_2.5_	2014 PM_2.5_
1	Australia	0.4342	1.0000	1.0000	1.0000	1.0000
2	Austria	0.1572	0.1609	0.1675	0.1671	0.1725
3	Belgium	0.1706	0.1913	0.1200	0.1541	0.1668
4	Canada	0.1934	0.2259	0.2306	0.2742	0.1329
5	Chile	0.0508	0.0545	0.0574	0.0590	0.0506
6	Czech Republic	0.0059	0.0064	0.0513	0.0475	0.0413
7	Denmark	1.0000	1.0000	1.0000	1.0000	1.0000
8	Estonia	0.0118	0.0136	0.0150	0.0138	0.0123
9	Finland	0.0965	0.2318	0.1420	0.3026	0.2885
10	France	1.0000	1.0000	1.0000	1.0000	1.0000
11	Germany	0.6625	0.6183	0.7461	0.8348	0.8288
12	Greece	0.1273	1.0000	1.0000	1.0000	1.0000
13	Hungary	0.0284	0.0299	0.0285	0.0290	0.0261
14	Iceland	1.0000	1.0000	1.0000	1.0000	1.0000
15	Ireland	1.0000	1.0000	0.2437	1.0000	0.3099
16	Israel	0.0640	0.0678	0.0661	0.0692	0.0633
17	Italy	0.5044	0.6028	0.5647	0.6091	0.5802
18	Japan	1.0000	1.0000	1.0000	1.0000	0.6126
19	Korea, Rep.	0.0250	0.0281	0.0278	0.0256	0.0251
20	Latvia	0.0064	0.0073	0.0081	0.0074	0.0067
21	Luxembourg	1.0000	1.0000	1.0000	1.0000	1.0000
22	Mexico	0.0299	0.0318	0.0295	0.0311	0.0310
23	Netherlands	1.0000	1.0000	0.2172	1.0000	0.2007
24	New Zealand	0.1340	0.1438	0.1489	0.1567	0.1488
25	Norway	1.0000	1.0000	1.0000	1.0000	1.0000
26	Poland	0.0100	0.0109	0.0110	0.0302	0.0107
27	Portugal	0.1171	0.1208	0.1193	1.0000	0.5055
28	Slovak Republic	0.0218	0.0232	0.0229	0.0232	0.0209
29	Slovenia	0.0150	0.0156	0.0136	0.0125	0.0109
30	Spain	0.4021	0.3627	0.3824	0.5364	0.4809
31	Sweden	0.9334	1.0000	1.0000	1.0000	1.0000
32	Switzerland	1.0000	1.0000	1.0000	1.0000	1.0000
33	Turkey	0.0136	0.0144	0.0155	0.0147	0.0132
34	United Kingdom	1.0000	1.0000	1.0000	1.0000	1.0000
35	United States	1.0000	1.0000	1.0000	1.0000	1.0000

**Table 9:** Energy, New Energy, CO_2_ and PM_2.5_ efficiency analysis.

No.	DMU	Energy	New Energy	CO_2_		PM_2.5_
1	Australia		The first year was less than 0.2, the other years were 1.	1 for 5 years	Last 4 years were 1 and the room for improvement was 0.	The room for improvement was 0.
2	Austria		Below 0.2, but continued to rise	Continue to rise to around 0.1	For more than five consecutive years, it was above 0.7, a slight decline in the last year	Slightly increased for five consecutive years, but all efficiency scores were below 0.2
3	Belgium		Fluctuated between 0.1 and 0.2	From 0.8 in 2010 to around 0.3 Then rising to around 0.5	1 for 5 years	Five years below 0.2 and falling
4	Canada		Fluctuated between 0.1 and 0.2	Below 0.1 and decreasing	1 for 5 years	Below 0.3, the previous four years rose but the last year dropped to around 0.1
5	Chile		Fluctuated around 0.1	Less than 0.1, then slightly rise	From 0.6 in the first year, down to 0.4 in the last year	Below 0.1, the first four years rose, the last year fell slightly
6	Czech Republic		From about 0 to about 0.1, and the last two years dropped slightly	Less than 0.1, then slightly rise	In 2010, 0.6 rose to 0.7 in 2011, and then fell to around 0.3.	Far below 0.1, and continued to decline
7	Denmark		1 for 5 years	1 for 5 years	1 for 5 years	1 for 5 years
8	Estonia		Less than 0.1, slightly rising	Close to 0	Five years around 0.2	Lower than 0.1
9	Finland		From 0.1 fluctuated to above 0.2	Below 0.1 and declining	From 1 in 2010, down to 0.6	From 0.2 in 2010 to 0.3 in 2014
10	France		1 for 5 years	1 for 5 years	1 for 5 years	1 for 5 years
11	Germany		Fluctuating around 0.8	Rose from around 0.3 in 2010 to nearly 0.8 in 2013, and dropped slightly to around 0.7 after 2014.	1 for 5 years	From about 0.6 in 2010 to 0.8
12	Greece		In 2010, it was 0.1, and after four years, it was 1	1 for 5 years	In 2010 it was 0.7 and then rose to 1	In 2010, it was about 0.1, and then rose to 1 in the last 4 years.
13	Hungary		Less than 0.1 and continued to drop slightly	Below 0.1 and decreasing	Fluctuated down to around 0.5 in 5 years	Below 0.1 and decreasing
14	Iceland		1 for 5 years	1 for 5 years	1 for 5 years	1 for 5 years
15	Ireland		In 2012 and 2014, it was about 0.1, and it was 1 in the other years	In 2012 and 2014, it was around 0.6, and was 1 in other years	In 2012, around 0.7, in 2014, 0.6, and is 1 in the other years	Less than 0.3 in 2012, less than 0.3 in 2014, and 1 in other years
16	Israel		Less than 0.1, slightly fluctuating, little change in five years	Below 0.1, but continued to rise slightly	Fluctuating between 0.6 and 0.7 for 5 consecutive years	Below 0.1, slightly fluctuating, but little change
17	Italy		From 0.4 in 2010 to above 0.8 in 2014	The sustained fluctuations from 0.6 in 2010 declined slightly. About 0.4 in 2014	0.9 in 2014, 1 in other years	Fluctuated between 0.5 and 0.6
18	Japan		Only in 2014 it fell to 0.6, 1 in other years	In 2014, it was around 0.9 and it was 1 in the other years	1 for 5 years	1 in the first four years and fell to 0.7 in the last year.
19	Korea, Rep.		Less than 0.1	From around 0.5 in 2010, it continued to drop to around 0.2.	Fluctuated at 0.7	Below 0.1 and decreasing
20	Latvia		Close to 0	Close to 0	Fluctuated between 0.6 and 0.7, a slight decline in the last year	Close to 0 and declining
21	Luxembourg		1 for 5 years	1 for 5 years	1 for 5 years	1 for 5 years
22	Mexico		Around 0.1	Around 0.1	Fluctuated between 0.7 and 0.8	Below 0.1
23	Netherlands		0.2 in 2012 and 2014, 1 in all other years	It was 0.6 in 2011, 0.4 in 2014, and 1 in other years.	1 for 5 years	In 2012 and 2014, only about 0.2, and 1 in other years
24	New Zealand		Around 0.1	Close to 0	Fluctuated between 0.8 and 0.9, fell to 0.8 in the last year	Fluctuated between 0.1 and 0.2, Slight decline in the last year
25	Norway		1 for 5 years	1 for 5 years	1 for 5 years	1 for 5 years
26	Poland		Below 0.1 and decreased	Below 0.1 and decreasing	Fluctuated between 0.5 and 0.6	Below 0.1, close to 0
27	Portugal		From 0.1 in 2010 to 1 in 2013, then dropped to around 0.4	From 2010, it was much lower than 0.1, rose to 1, and then dropped to around 0.2.	From 0.7 to 1 in 2013, a slight decline in 2014	From 2010 to 2012, around 0.1, 2013 to 1, 2014, drops to 0.5.
28	Slovak Republic		Below 0.1	Below 0.1 and declining	Fluctuated between 0.4 and 0.5	Below 0.1
29	Slovenia		Below 0.1 and declining	Below 0.1 and fluctuating down	Fluctuated between 0.5 and 0.6	Close to 0
30	Spain		Continued to rise from 0.3 to above 0.4	Fluctuated between 0.1 and 0.2	1 for 5 years	Fluctuated between 0.3 and 0.5
31	Sweden		Above 0.6 in 2010 continued to rise to 1	1 for 5 years	1 for 5 years	In 2010, 0.9, and 1 in the other years
32	Switzerland		1 for 5 years	1 for 5 years	1 for 5 years	1 for 5 years
33	Turkey		Below 0.1	Rising but all below 0.1	Fluctuated between 0.8 and 0.9	Below 0.1 close to 0
34	United Kingdom		1 for 5 years	1 for 5 years	1 for 5 years	1 for 5 years
35	United States		1 for 5 years	1 for 5 years	1 for 5 years	1 for 5 years
